# Critical Impact of Human Amniotic Membrane Tension on Mitochondrial Function and Cell Viability In Vitro

**DOI:** 10.3390/cells8121641

**Published:** 2019-12-15

**Authors:** Laura Poženel, Andrea Lindenmair, Katy Schmidt, Andrey V. Kozlov, Johannes Grillari, Susanne Wolbank, Asmita Banerjee, Adelheid Weidinger

**Affiliations:** 1 Ludwig Boltzmann Institute for Experimental and Clinical Traumatology, AUVA Research Center, Donaueschingenstraße 13, 1200 Vienna, Austria; laura.pozenel@trauma.lbg.ac.at (L.P.); andrey.kozlov@trauma.lbg.ac.at (A.V.K.); johannes.grillari@trauma.lbg.ac.at (J.G.); susanne.wolbank@trauma.lbg.ac.at (S.W.); adelheid.weidinger@trauma.lbg.ac.at (A.W.); 2 Austrian Cluster for Tissue Regeneration, 1200 Vienna, Austria; Andrea.Lindenmair@o.roteskreuz.at; 3 Ludwig Boltzmann Institute for Experimental and Clinical Traumatology, AUVA Research Center, Garnisonstraße 21, 4020 Linz, Austria; 4 Medical University of Vienna, Center for Anatomy and Cell Biology, Division of Cell and Developmental Biology, Schwarzspanierstraße 17, 1090 Vienna, Austria; katy.schmidt@meduniwien.ac.at; 5 University of Natural Resources and Life Sciences Vienna, Department of Biotechnology, Muthgasse 18, 1190 Vienna, Austria

**Keywords:** apoptosis, human amniotic membrane, mitochondrial cell death, BAX, BCL-2, tensile strength

## Abstract

Amniotic cells show exciting stem cell features, which has led to the idea of using living cells of human amniotic membranes (hAMs) in toto for clinical applications. However, under common cell culture conditions, viability of amniotic cells decreases rapidly, whereby reasons for this decrease are unknown so far. Recently, it has been suggested that loss of tissue tension in vivo leads to apoptosis. Therefore, the aim of this study was to investigate the effect of tissue distention on the viability of amniotic cells in vitro. Thereby, particular focus was put on vital mitochondria-linked parameters, such as respiration and ATP synthesis. Biopsies of hAMs were incubated for 7–21 days either non-distended or distended. We observed increased B-cell lymphoma 2-associated X protein (BAX)/B-cell lymphoma (BCL)-2 ratios in non-distended hAMs at day seven, followed by increased caspase 3 expression at day 14, and, consequently, loss of viability at day 21. In contrast, under distention, caspase 3 expression increased only slightly, and mitochondrial function and cellular viability were largely maintained. Our data suggest that a mechano-sensing pathway may control viability of hAM cells by triggering mitochondria-mediated apoptosis upon loss of tension in vitro. Further studies are required to elucidate the underlying molecular mechanisms between tissue distention and viability of hAM cells.

## 1. Introduction

Despite the over a century old tradition of using the human amniotic membrane (hAM) successfully for tissue regeneration in clinics [1,2,3,4,5], properties of the hAM are still subject of research.

The hAM starts to develop around day 7.5 of human gestation, far earlier than the formation of the three germ layers [6]. Forming the amniotic cavity, the hAM expands during the course of pregnancy and is supposed to rupture at term. It is usually discarded after childbirth, and, although of embryonic origin, the use and application of hAMs does not raise ethical issues. The hAM can be classified into different sub-regions. While both amniotic membranes are attached to the chorion, placental amnion (and chorion) covers the placenta, and reflected amnion (and chorion) is located opposite it. After preparation, the hAM is a thin, flexible and almost translucent membrane, harboring two vital cell populations. Human amniotic epithelial cells (hAECs) form a layer that, in vivo, faces the fetus, and is in direct contact with the amniotic fluid. Underneath, human amniotic mesenchymal stromal cells (hAMSCs) are embedded in a layer of extracellular matrix. Both cell populations have been proven to have stem cell characteristics, such as the ability to differentiate into lineages of all three germ layers in vivo and in vitro [7,8,9,10]. Furthermore, the cells express markers of pluripotency, an otherwise solely embryonic feature [11]. Properties of the hAM have been extensively described, as it is known to be anti-inflammatory [12,13,14,15] and immune-modulatory [14,15]. Moreover, remarkably, no substantial immune reactions upon application have been reported. We and others, furthermore, showed significantly different properties of cells of placental and reflected amnion in previous studies [16,17,18,19].

Up until the turn of the century, the hAM has normally been used in a denuded or decellularized form, making use of the composition of its extracellular matrix (reviewed in [5]). With increasing evidence of the stem cell properties of hAECs and hAMSCs, using hAMs with their original vital cell populations for tissue regeneration has come more and more into focus (reviewed in [5]). In other tissues and cell types, in recent years, researchers have concentrated on mitochondria in particular, as it has been shown that functional mitochondria are required to support tissue regeneration processes [20,21,22]. While beneficial properties of amniotic cells have been known for more than two decades, sustaining cellular viability of the hAM remains a challenge. For example, cryopreservation of hAMs under conditions reported does not result in any viable cells [23]. However, storage under common cell culture conditions is also not applicable, since several studies have shown decreasing cellular viability of the hAM [9,10,24]. To our knowledge, reasons for this rapid decrease of viability in vitro are not known so far.

However, in vivo, net loss of extracellular matrix [25] and apoptosis of hAECs are involved in the mechanisms leading to rupture of the membranes at term (reviewed in [26]). Parry therefore suggested that apoptosis of amniotic cells in vivo is probably a consequence of loss of tissue tension [26]. Of note, in vivo, the hAM is distended by a factor of 1.75 [27], a tensile strength that, ex vivo, is no longer existent. We therefore hypothesized that for in vitro culture of hAM, tensile strength plays an important role in the maintenance of cellular viability and that mitochondria play a critical role in this process.

The aim of this study was to clarify whether tissue distention controls apoptosis in a mitochondria-dependent manner and whether it can impact the viability of hAMs. To achieve this aim, we examined cellular viability, mitochondrial activity and activation of apoptotic pathways in distended compared to non-distended (floating) hAM samples in culture.

## 2. Materials and Methods

### 2.1. Preparation of the Human Amniotic Membrane (hAM)

Human placentae from caesarean sections were collected with informed consent of the patients and approval of the local ethical commission (Ethikkommission des Landes Oberösterreich, 21 May 2014), in accordance to the Declaration of Helsinki. Placentae were transported within 4 h in 500 mL Ringer solution. Placentae from caesarean sections of premature deliveries, emergency caesarean sections and placentae with detached amniotic membranes were excluded from the study. Placental (P) and reflected (RA) regions were separated, the hAM was peeled off the chorion and washed with cold phosphate-buffered saline (PBS).

### 2.2. Cultivation of hAM Samples

**Figure 1:**
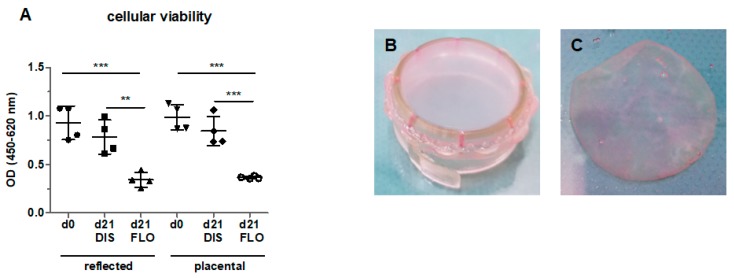
Cellular viability of reflected and placental amnion in fresh biopsies (day 0), biopsies mounted on CellCrown™ inserts (DIS), and biopsies kept floating (FLO) under common cell culture conditions for 21 days (**A**). Viability was measured with the EZ4U assay. Mean ± standard deviation (SD), n = 4 (donors). Samples of human amniotic membrane distended on CellCrown™ inserts (in 6 well plates) (**B**), and non-distended (“floating”) (**C**) at day 0. DIS: distended biopsies; FLO: floating biopsies; OD: optical density. **p < 0.01, ***p < 0.001.

For tissue distention, fresh hAM was mounted on CellCrown™ inserts (Scaffdex, Tampere, Finland) (Figure 1B) and incubated in Dulbecco′s Modified Eagle′s Medium (DMEM) supplemented with 10% fetal calf serum (FCS), 1% l-glutamine and 1% penicillin/streptomycin (“culture medium”) at 37 °C, a humidified atmosphere and 5% CO_2_. On the day of measurement or sample freezing, biopsies of 8 mm diameter of distended samples were punched. Non-distended (floating) samples (Figure 1C) were kept under the same conditions. All samples were measured at day 0 and incubated for 7 days (B-cell lymphoma 2-associated X protein (BAX), B-cell lymphoma (BCL)-2), 14 days (mitochondrial morphology, caspase 3), or 21 days (mitochondrial membrane potential, mitochondrial respiration, ATP concentration). The culture medium was changed twice weekly.

### 2.3. Cell Viability Assay

Cell viability of hAM biopsies (8 mm diameter) was quantified with the EZ4U—Cell Proliferation and Cytotoxicity Assay (Biomedica, Vienna, Austria). The assay was performed according to the manufacturer’s protocol. Briefly, the substrate solution was diluted 1:10 in DMEM without phenol red supplemented with 1% l-glutamine (Sigma-Aldrich, St. Louis, MO, USA). Biopsies were added to the solution and incubated for 3 h 45 min at 37 °C and 5% CO_2_. Plates were shaken for 15 min and the optical density (OD) was measured with a microplate reader (BMG Labtech, Polarstar Omega, Ortenberg, Germany) at 450 nm with 620 nm as reference. n = 4 (biological replicates).

### 2.4. Laser Scanning Confocal Microscopy

hAM samples were placed in 2-well chambered cover glass (Nunc™ Lab-Tek™, St. Louis, MO, USA) and stained with mitochondrial membrane potential sensitive fluorescent dye (500 nM tetramethylrhodamin-methylester (TMRM; VWR, Radnor, PA, USA (excitation/emission: 543 nm/585 nm)) for 45 min at 37 °C and 5% CO_2_. Imaging was performed with an inverted confocal microscope (LSM510, Carl Zeiss, Oberkochen, Germany). Image analysis (mean fluorescence) was performed with ZEN2009 Software (release version 6.0 SP2; Carl Zeiss). n = 2–3 (biological replicates).

### 2.5. High Resolution Respirometry

Mitochondrial respiratory parameters were monitored using high resolution respirometry (Oxygraph-2k, Oroboros Instruments, Innsbruck, Austria). Mitochondrial ROUTINE respiration, reflecting total mitochondrial oxygen consumption, was measured by incubating 14 hAM biopsies (8 mm diameter) in DMEM at pH 7.2 and 37 °C. For details, see Supplementary Material. Mitochondrial states were calculated as the negative time derivative of oxygen concentration (rate of oxygen uptake), and corrected for non-mitochondrial respiration (myxothiazol, 1 µM). Data were calculated in µM O/min/14 biopsies and are displayed in percent of placental amnion at day 0. n = 4 (biological replicates).

### 2.6. ATP Measurement

Liquid nitrogen frozen hAM biopsies (8 mm diameter) were homogenized in Precellys tubes with ceramic beads (Keramik-Kit 1.4 mm Peqlab VWR, USA) in a ball mill (CryoMill MM301, Retsch, Haan, Germany) with 500 µL of Tris-HCl buffer (20 mM Tris, 135 mM KCl, pH 7.4). Boiling buffer (400 µL of 100 mM Tris/4 mM EDTA, pH 7.75) was added to 100 µL hAM homogenate, incubated for 2 min at 100 °C and centrifuged at 1000× *g* for 2 min. ATP measurements were performed with the ATP Bioluminescence Assay Kit CLS II (Roche, Basel, Switzerland) in accordance with the manufacturer’s protocol using luciferase reagent with Lumat LB 9507 (Berthold, Bad Wildbad, Germany). For details, see Supplementary Material. n = 4 (biological replicates).

### 2.7. Histology

Amnion biopsies were fixed for 24 h in 4% formalin and samples were embedded in paraffin. Immunohistochemistry against caspase 3 was performed with an anti-cleaved caspase 3 antibody 1:100 (Cell Signaling Technology, Danvers, MA, USA). Immunohistochemical negative controls were performed by replacing the primary antibody with buffer. Immunohistochemical sections were quantified with ImageJ software (National Institutes of Health, version 1.51j8, Bethesda, MD, USA). n = 3 (biological replicates).

### 2.8. Transmission Electron Microscopy

Biopsies were fixed with 2.5% glutaraldehyde and 2% paraformaldehyde for 2–3 h at room temperature and post-fixed with 1% OsO_4_ in 0.1 M cacodylate buffer. Dehydration and embedding in Epon resin were carried out according to standard protocols. Sections (70 nm) were contrasted with 2% uranyl acetate. Images were acquired with an electron microscope (Tecnai20, FEI Europe, Eindhoven, Netherlands) equipped with a 4K EagleCCD camera and processed with Adobe Photoshop. n = 2 (biological replicates).

### 2.9. Reverse-Transcription Quantitative PCR Analysis

Samples of hAM biopsies (8 mm diameter) were snap-frozen in liquid nitrogen and kept at −80 °C until further analysis. Total RNA extraction, mRNA reverse transcription and qPCR were performed by TAmiRNA GmbH (Vienna, city, Austria). n = 3 (biological replicates).

Total RNA extraction: total RNA was extracted from 10 amnion biopsies (8 mm diameter) using the miRNeasy Mini Kit (Qiagen, Hilden, Germany). Tissue was homogenized with 700 µL Qiazol; following incubation at room temperature for 5 min, 140 µL chloroform was added to the lysates, which were incubated for 3 min at room temperature and centrifuged at 12,000× *g* for 15 min at 4 °C. Precisely 350 µL of the upper aqueous phase was transferred to a miRNeasy mini column, and RNA was precipitated with 525 µL ethanol followed by automated washing with RPE and RWT buffer in a Qiacube liquid handling robot. Finally, total RNA was eluted in 30 µL nuclease free water and stored at −80 °C until further analysis.

mRNA reverse transcription and qPCR (RT-qPCR): messenger RNA quantification was performed using the TATAA Grandscript cDNA synthesis and SYBR Grandmaster mix kit (TATAA Biocenter, Göteborg, Sweden). Total RNA (500 ng) was used for reverse transcription and all steps were carried out according to recommendations by the manufacturer. PCR amplification was performed in a 96 well format in a Roche LC480 II instrument (Roche, Mannheim, Germany) with the following settings: 95 °C for 30 s followed by 45 cycles of 95 °C for 5 s, 63 °C for 15 s and 72 °C for 10 s and subsequent melting curve analysis. To calculate the cycle of quantification values (Cq-values), the second derivative method was used. Cq-values were subsequently normalized to the geometric mean of glyceraldehyde 3-phosphate dehydrogenase (GAPDH), beta-actin (ACTB), hypoxanthine phosphoribosyltransferase (HPRT1) and ubiquitin C (UBC) mRNA levels, by subtracting the gene of interest Cq-value from the respective geometric mean of the four references. Primer sequences of BAX and BCL-2 used for mRNA reverse-transcription quantitative PCR analysis are shown in Table S1.

### 2.10. Statistical Analysis

Data were analyzed using GraphPad Prism software (GraphPad Software 5.01, San Diego, CA, USA) by one-way ANOVA followed by Bonferroni post hoc test. In all tests, n (sample size) represents biological replicates (donors). Results are presented as mean ± SD. Level of significance was set at 0.05 and is indicated as *p < 0.05, **p < 0.01, or ***p < 0.001.

## 3. Results and Discussion

All tests were performed with reflected and placental amnion samples, as we and others already have shown significant differences between reflected and placental amnion [16,17,18,19].

### 3.1. Cellular Viability Strongly Decreased in Floating hAM Samples

In order to test hAM viability in vitro, biopsies kept under floating conditions for 21 days were tested for cell viability with the EZ4U assay. Indeed, cell viability significantly decreased in reflected and placental amnion compared to fresh biopsies at day 0 (Figure 1A), confirming that standard cell culture conditions do not sufficiently maintain cell viability. This is in line with previous studies, where loss of viability was already observed [9,10,24]. Interestingly, in these studies, cells in non-distended hAMs remained viable under osteogenic [9], chondrogenic [24] or Schwann cell-like cell differentiation conditions [10]. This could mean that under differentiation conditions, cells may receive signals that sustain cell viability.

To test our hypothesis (whether mechanical tissue distention could prolong cellular viability and preserve mitochondrial function in in vitro culture), we incubated hAM biopsies for 21 days under two different conditions. For distended samples, the hAM was mounted on CellCrown™ inserts in order to expose the hAM to tensile strength (Figure 1B). For non-distended biopsies, we kept the biopsies “floating” in culture medium (Figure 1C). In contrast to floating biopsies, biopsies mounted on CellCrown™ inserts for 21 days showed no loss of cellular viability (Figure 1A). It has to be taken into account that in floating samples, cells still have close cell–cell contact, however, no mechanical tension on the tissue is present.

### 3.2. Mitochondrial Membrane Potential Drastically Decreased in Floating hAM Samples

**Figure 2:**
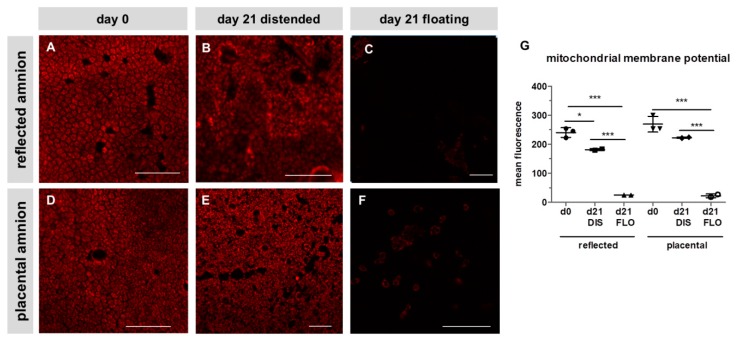
Mitochondrial membrane potential of reflected (**A**,**B**,**C**) and placental (**D**,**E**,**F**) amnion. Mitochondrial membrane potential (red) was stained with tetramethylrhodamin-methylester (TMRM; 500 nM) at day 0 (**A**,**D**) and at day 21 in biopsies cultivated while mechanically stretched (DIS; **B**,**E**) or kept floating (FLO; **C**,**F**). Imaging was performed with an inverted confocal microscope (LSM510, Zeiss, excitation/emission 543 nm/585 nm). Image analysis was performed with Zeiss ZEN2009 software (**G**). Mean ± standard deviation (SD), n = 3 (donors); representative images of one donor. Scale bar: 100 µm. DIS: distended biopsies; FLO: floating biopsies. Level of significance is indicated as *p < 0.05, ***p < 0.001

In order to see if mitochondria play a role in this strong decrease of cell viability, mitochondrial membrane potential was visualized after 21 days. Membrane potential was slightly decreased in distended reflected amnion (Figure 2B,G) compared to fresh hAM at day 0 (Figure 2A,G). Distended placental amnion (Figure 2E,G) at day 21 did not show any significant decrease compared to fresh biopsies at day 0 (Figure 2D,G). In contrast, mitochondrial membrane potential was drastically reduced in both reflected amnion (Figure 2C,G) and placental amnion (Figure 2F,G) under floating conditions after 21 days, compared to day 0 (Figure 2A,D,G) or distended biopsies (Figure 2B,E,G). Such changes in mitochondrial membrane potential can be connected to loss of cell viability [28].

### 3.3. Mitochondrial Respiration and ATP Concentration Strongly Decreased in Floating hAM Samples

**Figure 3:**
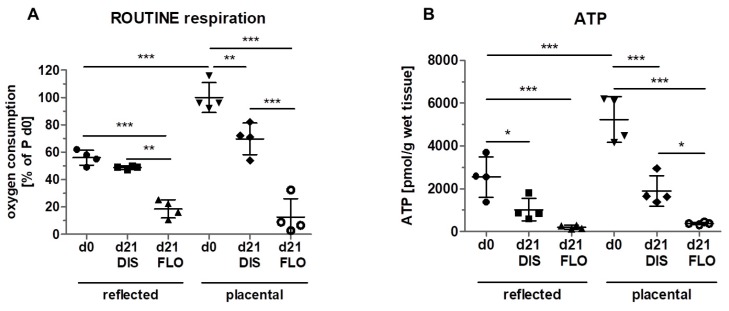
Measurement of mitochondrial activity. (**A**) Mitochondrial ROUTINE respiration (total mitochondrial oxygen consumption) was measured with high resolution respirometry (Oxygraph 2k, Oroboros Instruments) in reflected and placental amnion. Oxygen consumption was determined in fresh biopsies (day 0) and at day 21 in biopsies cultivated while mechanically stretched (DIS) or kept floating (FLO). Measurement of ATP levels. (**B**) ATP levels were determined using the ATP Bioluminescence Assay Kit CLS II (Roche) in fresh biopsies (day 0) and at day 21 in biopsies cultivated while mechanically stretched (DIS) or kept floating (FLO) in reflected and placental amnion. Mean ± SD, n = 4 (donors). P: placental amnion; DIS: distended biopsies; FLO: floating biopsies. Level of significance is indicated as *p < 0.05, **p < 0.01, ***p < 0.001.

Since mitochondrial membrane potential does not necessarily reflect mitochondrial activity, we determined mitochondrial ROUTINE respiration, a measure for total mitochondrial oxygen consumption. In fresh biopsies (day 0), significantly higher ROUTINE respiration was detected in placental compared to reflected amnion (Figure 3A). This is in line with a previous publication of our group [18]. No difference was observed between day 0 and distended reflected amnion at day 21. Placental amnion biopsies showed an approximately 30% lower ROUTINE respiration at day 21 compared to day 0. This is insofar interesting, as it has been shown that regarding mitochondrial activity, placental amnion is also much more responsive to changes in the microenvironment compared to reflected amnion [29]. In that study, responsiveness of placental amnion to inhibition of ATP synthase was much more pronounced. Furthermore, human amniotic mesenchymal stromal cells of placental amnion were found to be more susceptible to changes in oxygen tension [30]. As expected, ROUTINE respiration of both regions (reflected and placental) dramatically decreased in floating biopsies after 21 days compared to day 0. ROUTINE respiration was also significantly lower in floating compared to distended biopsies in reflected amnion. This effect was again even more pronounced in placental amnion (Figure 3A). The reasons for the repeatedly observed differences between reflected and placental amnion are still not known. We believe that the different anatomical locations of the hAM (one covering the placenta, the other opposite it) influence its properties. It is likely that during pregnancy, the two different amniotic sub-regions have different biological functions. Of note, regarding mechanical forces, the rupture of membranes at term takes place in the zone of altered morphology, an area within the reflected amnion [31].

The drastic decrease of mitochondrial oxygen consumption in floating samples of both amniotic regions, together with the massive loss of mitochondrial membrane potential, indicate severe mitochondrial dysfunction. We assume that this dysfunction is the prerequisite for the loss of cellular viability displayed in Figure 1.

Next, we wanted to see whether the changes in mitochondrial respiration also have an effect on ATP levels (Figure 3B). The results showed a similar pattern to the results of ROUTINE respiration. ATP concentrations at day 0 were also higher in placental amnion compared to reflected amnion, which is in line with a previous publication [29]. Distended biopsies after 21 days showed lower ATP concentrations compared to day 0 in both regions. Biopsies kept floating for 21 days showed a strong decrease in ATP concentrations in both regions (reflected and placental) compared to day 0. Floating biopsies of placental amnion showed lower ATP concentrations compared to distended biopsies. These data again indicate mitochondrial dysfunction.

Taken together, floating biopsies seem to have lost most of their viability, whereas in distended biopsies, viability could be sustained to a large extent. The question arose whether the loss of cellular viability was due to apoptosis or necrosis. The very low levels of ATP contradict apoptosis, since induction of apoptosis requires ATP [32,33]. We, however, measured ATP concentrations at a time point at which most of the cells had already lost their viability, meaning that the process of cell death had already been executed. In order to investigate whether apoptosis was involved in the initiation of cell death at an earlier time point, we shortened the incubation period and incubated non-distended and distended amnion biopsies for only 14 days.

### 3.4. Caspase 3 is Strongly Upregulated in Floating hAM Samples

**Figure 4:**
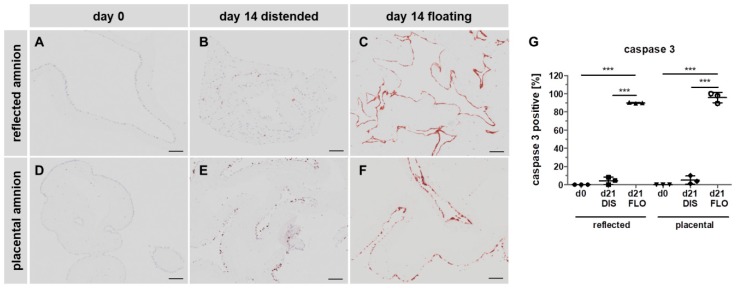
Caspase 3 immunohistochemical staining. Histological sections of reflected (**A**,**B**,**C**) and placental (**D**,**E**,**F**) amnion at day 0 (**A**,**D**) and at day 14 in biopsies cultivated while mechanically stretched (**B**,**E**) or kept floating (**C**,**F**) were stained for the expression of apoptosis marker caspase 3 (brown). Image analysis was performed with ImageJ software (**G**). Mean ± SD, n = 3 (donors); representative images of one donor. Scale bar: 100 µm. DIS: distended biopsies; FLO: floating biopsies. Level of significance is indicated as ***p < 0.001.

We first subjected paraffin-embedded hAM biopsies to immunohistochemistry staining with anti-cleaved caspase 3 antibodies. As expected, in fresh biopsies (day 0), no caspase 3 positive cells were detected in either amniotic region (Figure 4A,D,G). After 14 days, in distended biopsies, scattered expression of caspase 3 was found in reflected (Figure 4B,G) and placental samples (Figure 4E,G). In contrast, in floating biopsies, expression of caspase 3 drastically increased in both regions (Figure 4C,F,G), compared to fresh biopsies (Figure 4A,D,G), and distended biopsies (Figure 4B,E,G), indicating the occurrence of apoptotic cell death [34].

### 3.5. Severe Loss of Mitochondrial Internal Structure under Floating Conditions

**Figure 5:**
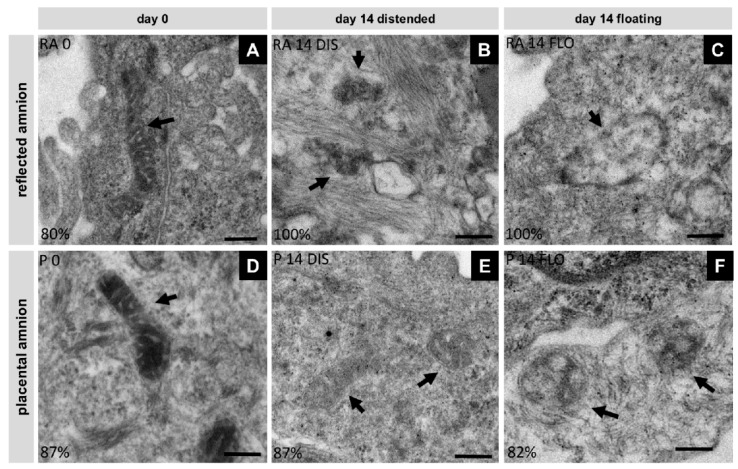
Changes in mitochondrial morphology were analyzed with transmission electron microscopy (Tecnai 20, FEI Europe, Eindhoven, Netherlands) in reflected (RA) (**A**,**B**,**C**) and placental (P) (**D**,**E**,**F**) amnion at day 0 (**A**,**D**) and at day 14 in biopsies cultivated while mechanically stretched (**B**,**E**) or kept floating (**C**,**F**), n = 2 (donors). Scale bar: 200 nm. Arrows indicate mitochondria. DIS: distended biopsies; FLO: floating biopsies.

In order to see if mitochondria are involved in the process of cell death, we investigated mitochondrial morphology using transmission electron microscopy. In fresh biopsies (day 0) of the hAM, mitochondria displayed well developed cristae within a strongly contrasted matrix in reflected (RA, Figure 5A) and placental (P, Figure 5D) hAM. At day 14, in distended samples, mitochondria of the placental region retained their cristae, however, the mitochondrial matrix appeared less dense (Figure 5E). Most of the mitochondrial cristae in distended reflected amnion were lost (Figure 5B). Floating samples showed mitochondria with severe loss of cristae and overall integrity in both regions (Figure 5C,F). These internal structural changes can be an indication for the onset of apoptosis [35,36].

### 3.6. Mitochondria-Linked Apoptotic Gene Expression was Upregulated within Seven Days

**Figure 6:**
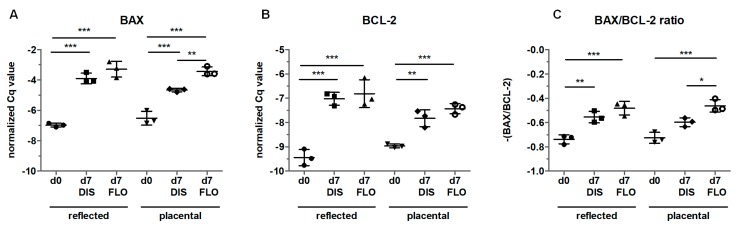
Gene expression of B-cell lymphoma 2-associated X protein (BAX) (**A**) and B-cell lymphoma (BCL)-2 (**B**) was determined by qPCR in fresh biopsies (day 0) and at day 7 in biopsies cultivated while mechanically stretched (DIS) or kept floating (FLO) in reflected and placental amnion. The results were normalized on the geometric mean of 4 different reference genes. The BAX/BCL-2 ratio is shown in (**C**). Mean ± SD, n = 3 (donors). DIS: distended biopsies; FLO: floating biopsies; Cq value: cycle of quantification value. Level of significance is indicated as *p < 0.05, **p < 0.01, ***p < 0.001.

In order to confirm that apoptosis was mitochondria-linked, we compared BAX and BCL-2 levels. Since we already observed strong upregulation in caspase 3 expression and changes in mitochondrial ultrastructure on day 14, we shortened the incubation time to seven days. Indeed, amnion samples showed higher gene expression of BAX and BCL-2 at day 7 in distended and floating biopsies in both regions compared to day 0 (Figure 6A,B). Moreover, floating placental amnion on day 7 showed significantly higher BAX expression compared to distended biopsies (Figure 6A). Calculating the BAX/BCL-2 ratio revealed that BAX levels in distended samples were higher compared to day 0, but this was only significant in reflected amnion (Figure 6C). As expected, the most pronounced effects were observed between day 0 and floating biopsies of day 7 for both regions (Figure 6C). Additionally, floating placental amnion had a higher BAX/BCL-2 ratio compared to distended biopsies. Although the increase in the BAX/BCL-2 ratio in distended samples also points to the onset of apoptosis, the low number of caspase 3 positive cells (Figure 4) showed that apoptosis was only partly initiated in distended samples. In floating samples, results of BAX/BCL-2 gene expression and caspase 3 immunohistochemistry indicate that the lack of tissue distention initiated mitochondria-mediated apoptosis. The results are in line with studies showing that the mitochondria-linked pathway via BAX is involved in the initiation of apoptosis in hAM in vivo [37,38], a crucial step that leads to the rupture of the membranes at term. Interestingly, it was also shown that chronic stretching of isolated human amniotic epithelial cells (hAECs) cultivated on flexible bottom cell culture plates increased the expression of pre-B cell colony-enhancing factor. This protected isolated hAECs from apoptosis [39], suggesting that distention can prolong cellular life span in vitro.

## 4. Limitations of the Study

A limitation of this study is that no specific set of inhibitors was used for this pathway.

## 5. Conclusions

Our data suggest that there is an unknown tension-driven mitochondrial pathway (TDMP), which may control viability of hAMs via triggering mitochondria-mediated apoptosis. This process starts with loss of tissue tension, followed by activation of TDMP, impairment of mitochondria, the release of mitochondrial apoptotic factors, induction of caspase 3-mediated apoptosis and the loss of viability of hAM cells. The presence of tissue distention prolongs the cellular life span of human amniotic membranes. Further studies are required to obtain a detailed time course of activation of apoptosis and to investigate the TDMP to shed more light on the hAM physiology. This knowledge will support optimization of hAM tissue cultures, as the distended hAM comes closer to the in vivo situation than non-distended samples.

## Supplementary Materials

The following are available online at https://www.mdpi.com/2073-4409/8/12/1641/s1, Supplementary Methods 1: Measuring mitochondrial oxygen consumption using high resolution respirometry. 2: ATP measurement using Bioluminescence Assay Kit CLS II. Table S1: Primer sequences used for mRNA reverse-transcription quantitative PCR analysis.
