# Supplementary material for: Critical Impact of Human Amniotic Membrane Tension on Mitochondrial Function and Cell Viability In Vitro
Source: Cells. 2019 Dec 15;8(12):1641. doi: 10.3390/cells8121641 (PMC6953074; doi:10.3390/cells8121641)
Supplement: Supplementary file 1 [file cells-08-01641-s001.pdf]

Supplementary Materials

1. Measuring Mitochondrial Oxygen Consumption using High Resolution Respirometry

Before starting the measurement with the polarographic oxygen sensor, 2.3 mL of cell culture medium was pipetted into the oxygraph chamber. The cell culture medium was stirred continuously (750 rpm) for 30 min at 37 °C using a magnetic stirring bar. Air calibration was performed after reaching a stable oxygen flux signal. Then, 14 hAM biopsies (8 mm diameter) were added to the oxygraph chamber and the chamber was closed by insertion of the stopper. The oxygen concentration was recorded automatically. After 10 min, myxothiazol (1 µM) was added, which inhibits the transfer of electrons from reduced ubiquinone (QH2) to the Rieske iron sulphur protein (Oroboros instruments, O2k manual titrations: SUIT protocols with mitochondrial preparations. *Mitochondr Physiol Network* **2019**, 9.12(19), 1-4

2. ATP Measurement using the Bioluminescence Assay Kit CLS II

After centrifugation, ATP measurements were performed in supernatants of hAM homogenates. The supernatants were transferred to a fresh glass tube and stored on ice until measurement. Luciferase reagent (100 µL) was added to 100 µL of samples/standards/blank by automated injection. Light emission measurement (bioluminescence; relative light units (RLU)) was started after a 1 s delay (Lumat LB 9507, Berthold, Bad Wildbad, Germany). Tris-HCl buffer was used as blank. ATP concentrations were calculated using an ATP standard curve.

Table S1. Primer sequences used for mRNA reverse-transcription quantitative PCR analysis.

Gene	Forward Primer	Reverse Primer
BCL-2	TTTGAGTTCGGTGGGGTCAT	TGACTTCACTTGTGGCCCAG
BAX	TGGCAGCTGACATGTTTTCTGAC	TCACCCAACCACCCTGGTCTT
